# Comprehensive Risdiplam Synthesis Overview: From Cross-Coupling Reliance to Complete Palladium Independence

**DOI:** 10.3390/molecules30224365

**Published:** 2025-11-12

**Authors:** Georgiy Korenev, Maxim B. Nawrozkij, Roman A. Ivanov

**Affiliations:** 1 Medicinal Biotechnology Department, Sirius University of Science and Technology, Olimpiyskiy Ave. 1, 354340 Sirius, Krasnodar Region, Russia; maxim.nawrozkij@gmail.com (M.B.N.); ivanov.ra@talantiuspeh.ru (R.A.I.); 2 Institute of Physiologically Active Compounds, Federal Research Center of Problems of Chemical Physics and Medicinal Chemistry, Russian Academy of Sciences, 142432 Chernogolovka, Moscow Region, Russia

**Keywords:** risdiplam, spinal muscular atrophy (SMA), imidazo[1,2-*b*]pyridazine, pyrido[1,2-*a*]pyrimidin-4-one, 4,7-diazaspiro[2.5]octane, heterocyclization approaches

## Abstract

Risdiplam is the first approved small-molecule therapy for spinal muscular atrophy (SMA), a severe, progressive neuromuscular disorder. In addition to its clinical significance, risdiplam is of a great interest for organic and medicinal chemistry due to its complex molecular architecture. Its structure incorporates three highly substituted heterocyclic fragments—imidazo[1,2-*b*]pyridazine, pyrido[1,2-*a*]pyrimidin-4-one, and 4,7-diazaspiro[2.5]octane—that serve as both versatile synthetic building blocks and critical pharmacophoric elements for drug design and discovery. The increasing scientific interest in risdiplam has led to numerous publications and patent applications that describe alternative synthetic methodologies. Recently, our group has also developed and introduced efficient, scalable manufacturing routes for the preparation of the target substance and the key intermediates of its synthesis. This mini-review systematically analyzes a plethora of risdiplam assembly strategies and synthetic approaches, covering developments from 2013 to the present.

## 1. Introduction

**Figure 1:**
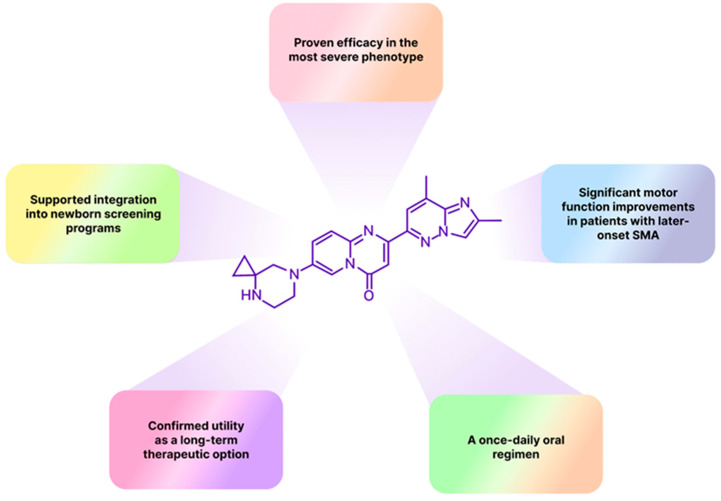
Outstanding therapeutic and pharmaceutical characteristics of risdiplam.

Spinal muscular atrophy (SMA), an autosomal recessive disorder that affects approximately 1 in 6000–10,000 newborns worldwide due to homozygous deletion of *SMN1* gene; it represents one of pediatric medicine’s most urgent therapeutic challenges. The condition is asymptomatic at birth but rapidly progresses to a neuromuscular disease that, in its most severe Type 1 form, typically manifests by three months of age and leads to death or permanent ventilator dependence within the first two years of life in the absence of disease-modifying treatment [1,2]. Risdiplam (Evrysdi) is an orally bioavailable *SMN2* splicing modifier for the treatment of 5q spinal muscular atrophy across the full clinical spectrum, from presymptomatic infants to adults [3]. The drug’s safety and efficacy were confirmed in a comprehensive clinical development program (Figure 1). In the pivotal FIREFISH study of risdiplam in infantile-onset SMA, 41% of treated infants achieved sitting without support for ≥5 s at 12 months, and 90% survived without permanent ventilation, establishing risdiplam’s efficacy in the most severe phenotype, where respiratory and nutritional complications typically limit therapeutic options [3,4]. The randomized, placebo-controlled SUNFISH trial has demonstrated in turn, statistically significant motor function improvements in patients with later-onset SMA (Types 2 and 3), with benefits most pronounced in younger participants [3,5].

The JEWELFISH study has provided safety and tolerability data for patients previously treated with other SMA therapies, demonstrating maintained tolerability over 24 months, with adverse event rates decreasing in the second year, supporting risdiplam’s utility as a long-term therapeutic option [6,7]. The RAINBOWFISH study showed that presymptomatic infants receiving risdiplam achieved age-appropriate motor milestones, which underpinned the FDA label expansion in May 2022 and catalyzed broader adoption of newborn screening programs for spinal muscular atrophy [8,9,10]. Finally, in September 2025, a nationwide real-world observational analysis provided results on the risdiplam use in adults. Patients with spinal muscular atrophy, predominantly Type 2 (40.4%) and Type 3 (47.4%), with a median age of 35.7 years and a median disease duration of 29.6 years, exhibited sustained, clinically meaningful functional gains that became most evident beyond 18 months of therapy. This data supports durable long-term treatment with risdiplam in adults and meaningfully closes the previously recognized evidence gap in the adult population treatment [11]. Overall, a once-daily oral dosing combined with efficacy in all SMA phenotypes positions risdiplam as a practical outpatient therapy across the disease spectrum.

**Figure 2:**
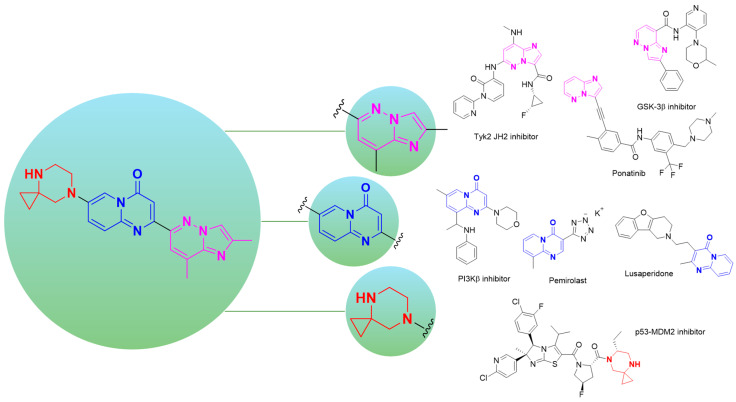
Highlighting three structural blocks within risdiplam— purple, imidazo[1,2-*b*]pyridazine; blue, pyrido[1,2-*a*]pyrimidin-4-one; red, 4,7-diazaspiro[2.5]octane —and a representative set of therapeutics and bioactive small molecules containing these building blocks.

However, beyond its exceptional clinical significance, risdiplam also showcases how structurally complex, highly substituted heterocyclic motifs can function as privileged scaffolds in contemporary drug discovery (Figure 2). The imidazo[1,2-*b*]pyridazine core exemplifies its versatility, most notably in the FDA-approved multi-kinase inhibitor ponatinib (Iclusig^®^) for chronic myeloid leukemia [12], and across diverse therapeutic programs including brain-penetrant GSK-3β inhibitors [13], orally active TYK2 JH2 inhibitors for autoimmune diseases [14] and various molecules with antimicrobial properties [15,16]. Recent structure–activity relationship studies highlight its capacity for systematic optimization through strategic substitution patterns, enabling precise modulation of potency, selectivity, and pharmacokinetic properties of the target substances [16].

The pyrido[1,2-*a*]pyrimidin-4-one motif in turn, represents one of the most pharmaceutically successful heterocyclic scaffolds, forming the core of numerous approved drugs including the anti-allergic agent pemirolast [17] and the antidepressant lusaperidone [18]. This framework continues to attract substantial interest from medicinal chemists, with applications spanning aldose reductase inhibitors [19], PI3K inhibitors for oncology [20], and CXCR3 inhibitors for inflammatory diseases [21]. Its synthetic accessibility via diverse methodologies and favorable drug-like properties position it as a valuable building block for pharmaceutical development [22].

Finally, the 4,7-diazaspiro[2.5]octane fragment reflects the growing recognition of spirocyclic systems as privileged building blocks in drug design [23]. While less explored than other spirocyclic frameworks, related diazaspiro systems have demonstrated utility in medicinal chemistry [24], including a promising p53–MDM2 interaction inhibitor [25]. Spirocyclic scaffolds, in general, offer distinct advantages including enhanced three-dimensionality, increased sp^3^ fraction, and conformational rigidity that enable exploration of previously inaccessible chemical space. This flexibility has been further enhanced by recent advances in novel strain-release spirocyclization methodologies, which have improved access to diverse diazaspiro frameworks, enabling their integration into screening libraries and lead optimization campaigns [26]. The widespread and successful utilization of these heterocyclic frameworks in targeted design of pharmacologically active compounds with a given type of biological activity unequivocally confirms that risdiplam serves not only as a breakthrough therapeutic agent but also as a valuable repository of privileged structural motifs for future drug discovery endeavors.

**Figure 3:**
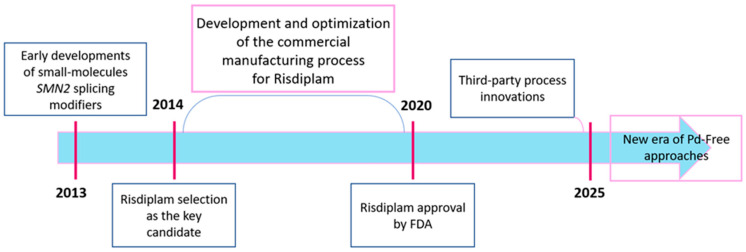
The timeline of risdiplam synthetic development and innovation.

The synthetic evolution of risdiplam itself, which also represents a compelling case study in modern pharmaceutical process development, encompassing over a decade of innovation from initial discovery through industrial optimization to contemporary academic refinements is also worth paying attention to. The timeline of synthetic innovation (Figure 3) reflects the collaborative efforts of diverse research communities worldwide—from industrial process chemists optimizing manufacturing efficiency to academic groups developing novel metal-free methodologies—each contributing unique perspectives and technical solutions to the synthetic challenges posed by risdiplam’s complex architecture.

**Figure 4:**
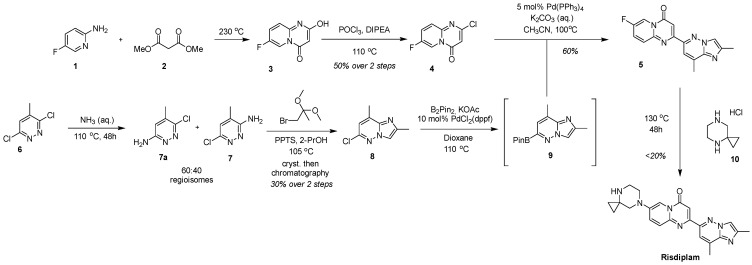
The originally developed scheme for the synthesis of risdiplam.

The original industrial synthetic scheme, documented in Roche’s foundational patents and process disclosures, established a multi-step palladium-catalyzed approach featuring borylation and Suzuki-type cross-coupling reactions to assemble the key heterocyclic fragments (Figure 4) [27,28]. This baseline methodology, while effective for establishing proof-of-concept and early clinical supplies, utilized multiple purification steps that presented scalability challenges for commercial manufacturing.

The industrial process evolution took around a decade, through late 2014 until early 2024, as evidenced by a growing patent landscape encompassing not only the original Roche intellectual property but also third-party process innovations claiming improved telescoped sequences, alternative protecting group strategies, and enhanced impurity control methodologies [29,30,31,32,33]. This period also witnessed the expansion of the patent landscape to include polymorph patents, formulation improvements, and combination therapy claims, reflecting the comprehensive intellectual property strategy surrounding a commercially successful pharmaceutical [34,35,36,37]. The most recent phase of synthetic development, exemplified by our group’s 2025 academic publications, has introduced paradigm-shifting methodologies that fundamentally challenge the metal-dependence of traditional approaches [38,39]. These innovations not only address cost and supply chain considerations inherent in palladium-based processes, but they also open up new avenues for synthetic exploration and optimization strategies.

This rich tapestry of synthetic evolution—spanning originator industrial chemistry, competitive process development, and cutting-edge academic innovation—has generated a substantial body of literature encompassing diverse strategic approaches, novel intermediates, and innovative methodologies. The accumulated knowledge base now warrants systematic analysis to distill key insights, evaluate synthetic efficiency trends, and identify emerging opportunities for further advancement. This mini-review aims to provide such a comprehensive assessment, leveraging our group’s expertise to critically evaluate the benefits and limitations of each approach while highlighting the most promising directions for future synthetic methodology development.

## 2. Advances, Limitations, and Major Challenges in Risdiplam Synthesis

**Figure 5:**
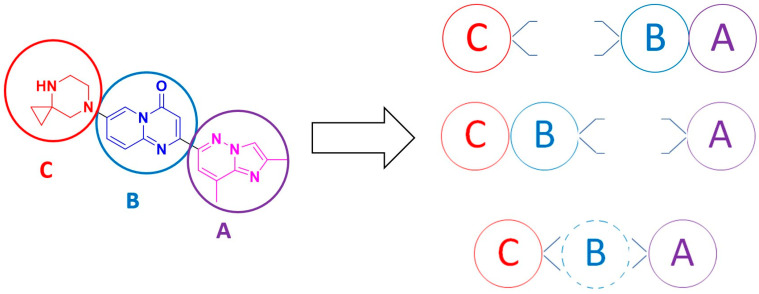
Structuring and visualization of three main synthetic approaches towards the risdiplam molecule.

Since the first experimental preparation of risdiplam [28], numerous synthetic pathways have been reported. These can be organized into three unequal classes, that differ in fragment preparation and in the sequence used to assemble the target molecule. Risdiplam scaffold can be virtually sliced into three previously defined building blocks, labeled from A to C, to facilitate navigation, structuring, visualization and examination of alternative assembly sequences (Figure 5).

### 2.1. C + BA-Strategies Towards the Target Molecule

**Figure 6:**
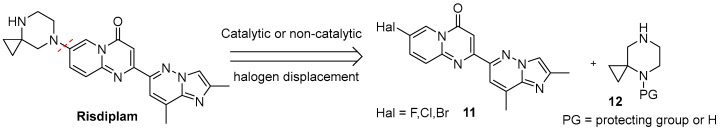
The first retrosynthetic disconnection (red dotted line) in risdiplam C + BA—synthetic strategies.

From a retrosynthetic standpoint, most routes of this kind, on the final stages rely on direct substitution of a leaving group at C-7 position in 2-(2,8-dimethylimidazo[1,2-*b*]pyridazin-6-yl)-7-halo-4*H*-pyrido[1,2-*a*]pyrimidin-4-one **11** with the *N*-4-protected or unprotected 4,7-diazaspiro[2.5]octan-7-amine moiety **12** (Figure 6). Depending on the halogen atom, the C–Hal displacement may proceed catalytically (typically for Cl- or Br-atoms) or in a catalyst-free manner (typically for F-atom). Pd-catalyzed substitutions of Cl and Br atoms are well established in related substrates and span a wide range of conditions and yields [40,41,42,43]. Nevertheless, they have seen limited adoption in risdiplam synthesis, likely because most known and well-described routes to risdiplam are already burdened with additional Pd-catalyzed cross-coupling steps, so it appears mainly as isolated examples in a few patents [44,45].

In contrast, the non-catalyzed substitution at C–F has been described repeatedly for risdiplam and its bioisosteric analogs. In the early phases of risdiplam development, substitutions of the fluorine atom for cyclic and spirocyclic amine moiety were initially conducted in high-boiling polar aprotic solvents (e.g., DMA, NMP) [46,47], then in DMSO with K_2_CO_3_ or triethylamine [48,49], and—once risdiplam had been successfully secured as the lead compound—in DMSO with DIPEA [27]. Notably, during the post-discovery optimization of the final step of such synthetic approaches, the focus has shifted from solvent/base selection to the deliberate choice of a protecting group on the incoming 4,7-diazaspiro[2.5]octane **12** fragment. It is also important to note that amine **12**, and its protected forms, are widely available with no restrictions from multiple commercial suppliers. It makes the development of routes from simpler precursors commercially unattractive and highly resource consuming. Accordingly, throughout this review, intermediate **12** and any of its derivatives are considered as readily available synthetic building blocks, or as intermediates, whose preparation from the unprotected/Boc-protected analogs is not expected to be challenging.

Different kinds of *N*-acyl protection of the spiroamine using formyl **12a** [50], (benzyloxy)carbonyl **12b** [33] or *tert*-butoxycarbonyl **12c** [39] provide superior chemo- and regioselectivity in comparison with utilization of the free amine **12d** [27], typically increasing the isolated yield of the substitution product (Table 1). Given broadly similar yields, Boc-protection is typically preferred owing to its mild cleavage conditions and the downstream ability to form an isolable risdiplam salt that can be readily converted to the free base.

**Figure 7:**
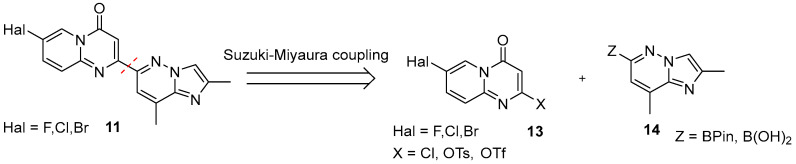
The second retrosynthetic disconnection (red dotted line) in risdiplam C + BA—synthetic strategies.

However, it is quite obvious, that the pivotal step on the path to the target molecule is the rapid, scalable route to the assembly of the key intermediate **11**. As a rule, it is prepared by Pd-complex catalyzed cross-coupling of 7-halo-4*H*-pyrido[1,2-*a*]pyrimidin-4-one fragment **13**, containing a good nucleofuge at C-2 position, and 2,8-dimethylimidazo[1,2-*b*]pyridazin-6-boronic acid (or its pinacol ester) **14** (Figure 7).

In contrast to the diversity seen in the terminal substitution step, converging the two fragments **13** and **14** to afford target **11** is comparatively uniform across reports and offers limited methodological variety.

**Figure 8:**
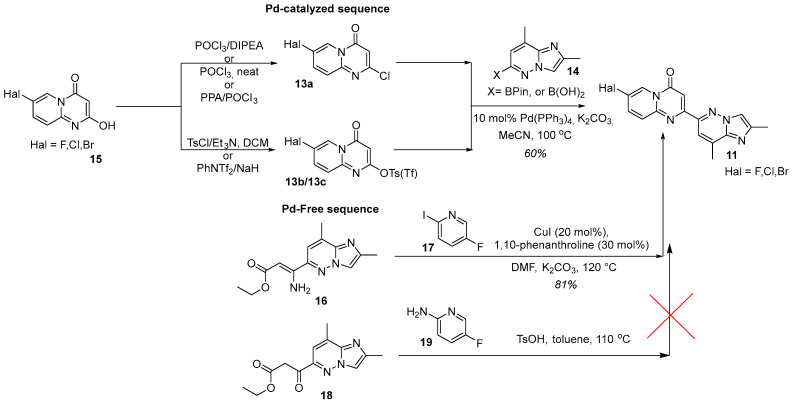
Pd-catalyzed sequence: standard C-2 activation strategies on the 7-halo-4*H*-pyrido[1,2-*a*]pyrimidin-4-one core **15** and conditions for construction of the key intermediate **11**; Pd-Free sequence: Cu(I)-catalyzed heterocyclization route and failed alternative approach to the same target intermediate **11**.

The pivotal operation is activation at C-2 position of the 7-halo-4*H*-pyrido[1,2-*a*]pyrimidin-4-one core **15** by deoxychlorination or *O*-acylation. For 7-fluoro derivatives, POCl_3_ with Hünig’s base commonly used [27,28,33,44,47,48,49,51]; neat POCl_3_ or the PPA/POCl_3_ system are also effective, particularly for 7-bromo and 7-chloro analogs [52,53,54,55]. When a better nucleofuge is required, *O*-tosylation (e.g., TsCl/Et_3_N in CH_2_Cl_2_) or triflation (e.g., PhNTf_2_/NaH) is employed [30,44] (Figure 8). The resulting activated intermediate **13a** or **13b/13c** then undergoes a standard Suzuki–Miyaura reaction with coupling-ready partner **14** in the presence of Pd(PPh_3_)_4_ and aqueous K_2_CO_3_ in MeCN at 100 °C, typically furnishing the desired product in ca. 60% yield [27,30,33]. Alternatively, the key intermediate **11** can be assembled without palladium catalysis, but via a Cu(I)-catalyzed heterocyclization between a 3-aminoacrylate **16** and 5-fluoro-2-iodopyridine **17** with 1,10-phenanthroline as the ligand of choice in the DMF/K_2_CO_3_ system at 120 °C [39] (Figure 8). It is worth noting that an easier pathway, based upon the cyclocondensation of 3-oxoester **18** and the proper 2-aminopyridine derivative **19** under catalyst-free conditions, although previously effective for closely related analogs [48], proved to be totally unsuccessful for risdiplam synthesis [39].

**Figure 9:**
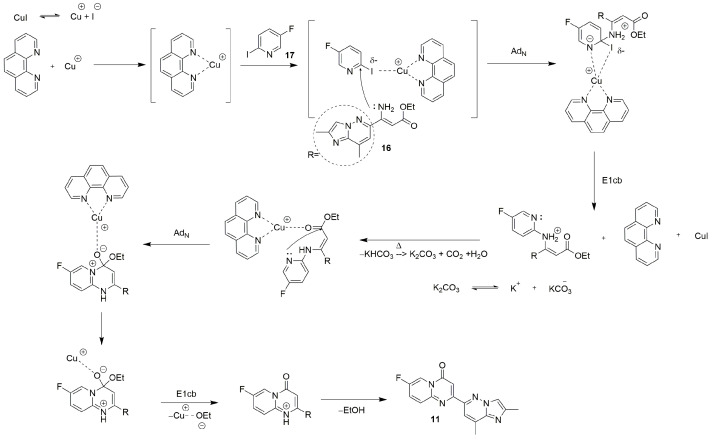
The plausible mechanism for the Cu(I)-catalyzed heterocyclization of intermediates **16** and **17** to obtain the key building block **11**.

The Pd-free heterocyclization described above, which within the chosen strategy furnishes the required block **11**, is both unique and nontrivial in the context of risdiplam synthesis. In contrast to standard, well-studied palladium-catalyzed cross-coupling reactions, the present transformation is empirically validated yet lacks a definitive description of mechanism. To clarify the underlying chemistry and to enable broader application in assembling related heterocyclic frameworks, we next propose a plausible mechanism for this heterocyclization (Figure 9).

Notably, 1,10-phenantroline appears to be better for this reaction, than the corresponding sterically hindered phosphine ligands. This fact is in good agreement with the data of Beletskaya and co-workers [56] and is opposite to the other published data [57].

While the final steps of risdiplam molecule construction include innovative and demanding operations, the true locus of the chemical challenge lies in accessing the requisite building blocks **14** and **15**, which enable the foregoing transformations. The synthesis of 7-halo-2-hydroxypyrido[1,2-*a*]pyrimidin-4-one precursors **15** can be readily accomplished through established Conrad-Limpach methodology variants—employing either thermal condensation of 2-amino-5-halopyridines with dialkyl malonates at elevated temperatures [28,44,47,48,58] or their reaction with malonyl dichloride [54,59] or bis-(2,4,6-trichlorophenyl)malonate under mild conditions [60,61,62]. Among the synthetic steps towards the target compound, this condensation demands particular attention.

While malonyl dichloride, with its potent reactivity, promises the gentlest conditions for this transformation [54,59], its hydrolytic instability and corrosive nature render it a capricious and challenging reagent to handle. Bis-(2,4,6-trichlorophenyl)malonate emerges as a compelling alternative to the troublesome acyl chloride, mirroring its mildness and delivering comparable yields [60,61,62] while boasting superior stability and diminished corrosivity. Here, the 2,4,6-trichlorophenoxy group proves a good nucleofuge—a linchpin of the “addition-elimination” mechanism that drives the reaction to fruition. Conversely, the allure of less expensive dialkyl malonates (dimethyl [47,48], diethyl [58], and di-*tert*-butyl [63]) fades under scrutiny, their application demanding harsh conditions due to the recalcitrance of methoxy, ethoxy, and especially *tert*-butoxy groups to anionoid separation. Indeed, the feasibility of employing di-*tert*-butyl malonate hangs in considerable doubt, its bulky *tert*-butyl groups exerting both a significant positive inductive effect and a formidable steric hindrance around the ester carbonyl, rendering nucleophilic attack a Herculean task. Tellingly, the lone documented instance of di-*tert*-butyl malonate’s use resides within a Roche patent detailing the synthesis of risdiplam, standing in stark contrast to the numerous reports showcasing the utility of its more amenable congeners.

**Figure 10:**
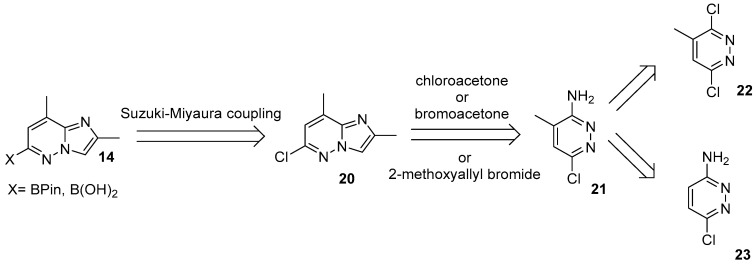
Retrosynthetic pathway of the key 2,8-dimethylimidazo[1,2-*b*]pyridazin-6-substituted building-block **14**.

The corresponding imidazo[1,2-*b*]pyridazine coupling partner **14**, in turn, poses significantly greater synthetic challenges due to its limited commercial availability and nonconventional substitution pattern. Even the first two retrosynthetic disconnections (Figure 10) toward intermediates of type **14** being quite simple, allow multiple practical implementations.

Typically, boronic acid (or its pinacol ester) **14** is obtained in an almost quantitative yield, without additional purification via Miyaura borylation of the corresponding 6-chloro-2,8-dimethylimidazo[1,2-*b*]pyridazine **20** with B_2_Pin_2_. Common catalyst/ligand systems include different options: PdCl_2_(dppf) [27,28,33,51,63,64,65], XPhos–Pd variants [66], and Pd(OAc)_2_ with PCy_3_ [30]. Direct routes to the free boronic acid in turn, employ both abovementioned PdCl_2_(dppf) or Pd_2_(dba)_3_/XPhos system [67,68,69,70,71,72].

However, despite the nature of the required boronic acid derivative, the irreplaceable starting material for each synthesis remains imidazo[1,2-*b*]pyridazine **20**. This compound is known for decades [73] and is recognized as an important building block for the synthesis of herbicides [74] and various pharmaceuticals. The most practical syntheses of it start from 3-amino-4-methyl-6-chloropyridazine **21**, followed by annulation with bromo- or chloroacetone, or with in situ–generated 2-methoxyallyl bromide [27,67,72,73,75,76].

It is worth noting that in virtually all reported routes to risdiplam, pyridazine **21** constitutes the principal bottleneck and the most problematic intermediate. The cornerstone of the syntheses of **21** is a poor regioselectivity of the chlorine substitution in 3,6-dichloro-4-methylpyridazine **22**, leading to a mixture of regioisomers together with the traces of a product of double substitution. These two regioisomers are markedly different in their melting point and can be, more or less, effectively separated either by chromatography on aluminum oxide [77] or by fractional recrystallization from ethanol [78,79]. In both cases the product is obtained by the reaction of **22** with ethanolic ammonia. Up to date, a number of modern protocols describe the use of aqueous ammonia instead for this purpose [27,51,80], but these approaches lead to the formation of the products of hydrolysis, as well, making the purification of the target compound accessible only via chromatographical techniques. Given the relatively harsh conditions, extended reaction times, and frequent reliance on chromatography, the quality of the final risdiplam—and its ability to satisfy pharmacopeial specifications—depends critically on the quality of the starting pyridazine **21**. These considerations argue for a fundamentally different strategy to access this building block.

**Figure 11:**
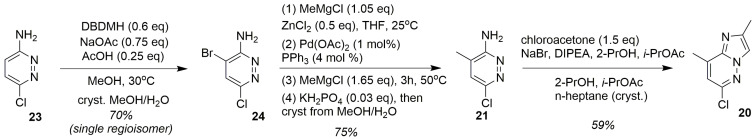
Optimized manufacturing process for key intermediate **20**.

In order to secure reproducible, chromatography-free access to pyridazine **21** synthesis without isomer separation, Roche devised an alternative strategy in the course of the route optimization campaign, which was ultimately adopted in the final commercial synthesis of risdiplam. The fundamental idea was to invert the order of the introduction of the substituents, beginning with commercially available 3-amino-5-chloro-pyridazine **23** as the starting material [30]. This approach enabled precise regiocontrol through sequential functionalization steps (Figure 11).

The initial bromination of **23** using elemental bromine provided only 40% yield, accompanied by significant handling and storage challenges. Systematic evaluation of alternative brominating agents led to the identification of 1,3-dibromo-5,5-dimethylhydantoin (DBDMH) as the optimal reagent for this purpose. Using DBDMH in a buffered NaOAc/AcOH (3:1) solution in methanol, followed by Na_2_SO_3_ quenching and crystallization, provided compound **24** as a single regioisomer in 70% yield with >99% purity on 200 kg scale [30]. The subsequent methylation via Negishi coupling appeared to be very tedious. Initial attempts using pyrophoric dimethylzinc were deemed unsuitable for large-scale operations. The resolution involved generating the organozinc reagent in situ from readily available ZnCl_2_ and methylmagnesium chloride (which was added in two portions). The first portion (1.05 eq) was to control methane formation and the second (1.65 eq) was to trigger the methylation. Careful temperature control (50 °C) and Pd/L ratio optimization (1:4) to minimize bis-methylation (<1%), rendered the desired compound **21** in an excellent yield. The addition of K_2_HPO_4_ was needed to precipitate any residual zinc and palladium salts, which were filtered off just before the crystallization of the pure product from the MeOH/H_2_O system. The final cyclization employed 2-chloroacetone, as was mentioned before, in the presence of Hünig’s base at 80 °C, with sodium bromide, serving as a reaction facilitator. Following comprehensive workup and dual crystallization from *i*-PrOAc/2-PrOH/*n*-heptane, intermediate **20** was obtained in >99.8% purity and 59% yield from compound **21** [30]. This systematic process development transformed a low-yielding, chromatography-dependent discovery route into a scalable, high-purity manufacturing process, demonstrating the critical importance of regioselectivity control and process optimization in pharmaceutical manufacturing.

Nevertheless, despite the outstanding achievements in preparing intermediate **20**, even the developed commercial process suffers from numerous drawbacks that complicate its technical implementation. The challenging palladium-catalyzed modified Negishi coupling, which is a key step in the synthetic scheme, presents several critical issues including stringent requirements for methane evolution control, rigorous removal of palladium residues, and management of *bis*-methylation side products. Combined with the modest overall yield of 31% for this sequence, these factors render the preparation of these intermediates as still a technically demanding challenge.

While the independent preparation of key building blocks introduces convergent elements into the synthetic strategy, the final risdiplam assembly proceeds fundamentally through a linear C + BA (Figure 5) sequence, creating exceptional opportunities for structural diversification in the design of related therapeutic compounds. This modular framework enables systematic modification of individual heterocyclic components, establishing a versatile platform for structure-activity relationship exploration and analog development.

### 2.2. CB + A and Convergent C + A (With the Formation of B During the Reaction) Approaches Toward Risdiplam

Contemporary risdiplam synthesis increasingly employs convergent schemes, exemplified by both Roche’s optimized commercial process and recent academic innovations that have redefined synthetic accessibility. These converging methodologies not only amplify manufacturing efficiency but also safeguard the vital flexibility needed for pioneering medicinal chemistry. This reflects a strategic metamorphosis from the nascent pathways of discovery to impeccably refined production processes, poised to meet both the rigors of commercial demand and the ever-evolving frontier of therapeutic innovation.

**Figure 12:**
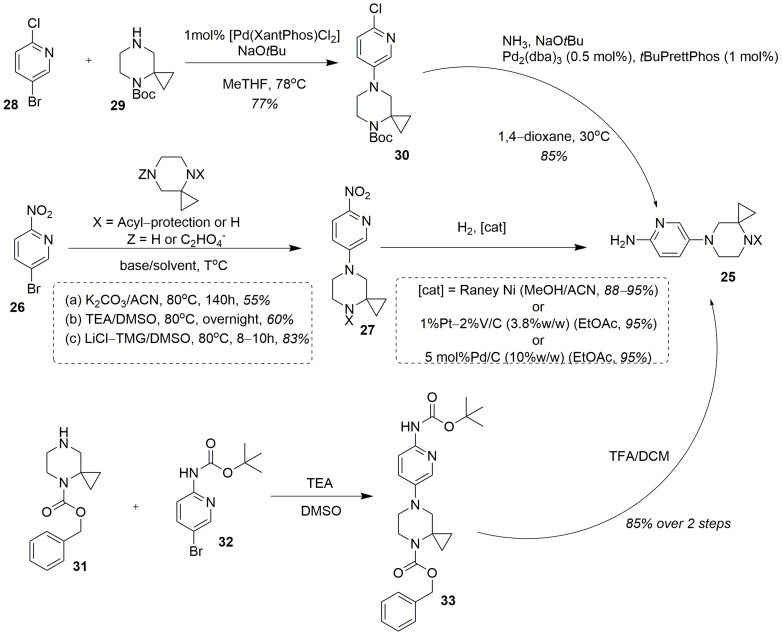
Three main synthetic approaches to the key intermediate **25**.

Besides the C+BA strategy, several convergent syntheses of risdiplam have been described and patented. The 5-(4,7-diazaspiro[2.5]octan-7-yl)pyridin-2-amine **25** turned out to be an important intermediate here. However, in most cases, it contained a protected imino-group [32,63,81]; there is also a sole exception of utilization of this amine in a free form as a key intermediate in this process [82]. Compound **25** can be prepared by a well-described two-step protocol. The first step is the amino-dehalogenation of 5-bromo-2-nitropyridine **26** with the proper spirocyclic amine, yielding the corresponding nitropyridine **27** in a free or acylated form. In most cases, a Boc-protected form of the spirocyclic amine has been used for this purpose [32,38,83,84]. The utilization of a free 4,7-diazaspiro[2.5]octane [85], however, seems doubtful due to regioselectivity issues, connected with the possible hetarylation of the two unequal nitrogen atoms in the structure of the spirocyclic amine. This reaction can be conducted either under metal-complex catalysis: for example, with Pd_2_(dba)_3_ in the presence of BINAP and Cs_2_CO_3_ in 1,4-dioxane [86], as well, as without it. In the latter cases the solvents of choice were DMSO [63,85,86] or MeCN [32], combined with triethylamine [83], potassium carbonate [32,85] or the “lithium chloride—tetramethylguanidine” system [30,63,84], as a base, giving the desired intermediate **27** (Figure 12).

The thus obtained nitro-compound **27** is then subjected to catalytic hydrogenation (in the presence of Raney nickel [85], platinum [32] or a palladium catalyst [38,81,86]) resulting in the key intermediate **25**. In the literature we can also find two synthetic protocols for the preparation of an intermediate of a kind, avoiding the use of 5-bromo-2-nitropyridine. One of them relies on a palladium-complex catalyzed amino-dehalogenation of 5-bromo-2-chloropyridine **28** with Boc-protected spiroamine **29**, followed by catalytic chlorine substitution for amino-group [30,63] (Figure 12). Alternatively, the similar Cbz-protected intermediate may be prepared by direct interaction of *tert*-butyl N-(5-bromopyridin-2-yl)carbamate **32** with 1-Cbz-protected spiroamine **31**, followed by acidic cleavage of the Boc-protective group [87]. However, this protocol also seems doubtful due to the absence of the electron-withdrawing group in *para*-position to the bromine atom, making it much more resistant to the uncatalyzed nucleophilic displacement.

**Figure 13:**
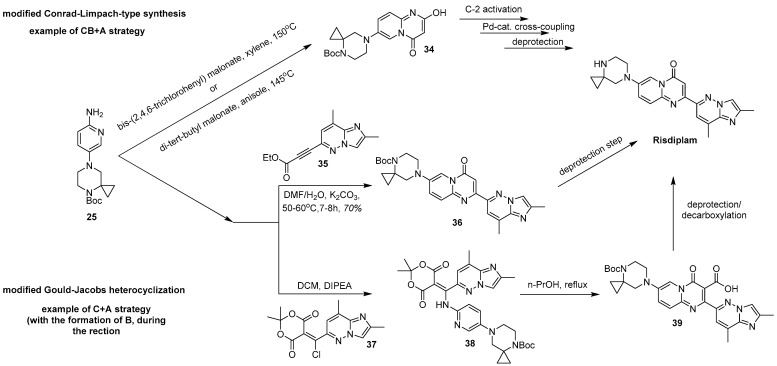
Two main strategies toward risdiplam in a convergent manner from the key intermediate **25**.

Next, regardless of the chosen synthetic way—the key intermediate **25** may be either introduced into a modified Conrad-Limpach-type synthesis with either *bis*-(2,4,6-trichlorophenyl) malonate [84,85] or di-*tert*-butyl malonate [63], or alternatively, it can undergo a modified Gould-Jacobs heterocyclization. In the first case, a *N*-protected 7-(4,7-diazaspiro[2.5]octan-7-yl)-2-hydroxy-4*H*-pyrido[1,2-*a*]pyrimidin-4-one **34**, which is a valuable building block for the further preparation of risdiplam, is formed. Then, the already mentioned *O*-tosylation [63,84] or deoxychlorination [85], followed by a palladium-complex catalyzed cross-coupling reaction, results in an *N*-protected form of risdiplam, which in turn, can be conveniently converted into the target compound (Figure 13).

A modified Gould-Jacobs reaction, in turn, is based upon the interaction of ethyl ester of 3-(2,8-dimethylimidazo[1,2-*b*]pyridazin-6-yl)propiolic acid **35** [31,82,88] or its synthetic surrogate: 5-[chloro(2,8-dimethylimidazo[1,2-*b*]pyridazin-6-yl)methylidene]-2,2-dimethyl-1,3-dioxane-4,6-dione **37** (prepared in situ from the corresponding acylated Meldrum’s acid and Vilsmeyer-Haack regent) [32,38]. This then leads to either risdiplam itself (after deprotection work-up) [82] or its *N*-protected carboxylated form **39 [32,38]** (Figure 13). The latter may be converted into the target compound upon an acidic reflux condition which is needed to afford the deprotection/decarboxylation step.

The main disadvantage of these two pathways is the low stability of both the propiolic acid derivative **35** and the intermediate **37**. The first one has a high tendency for oligo- and polymerization due to the presence of a basic nitrogen together with an activated acetylenic bond in the same molecule. The second one is endowed with a highly reactive chlorine atom in its structure, capable of different substitution reactions. Moreover, it is known from the literature that the heterocyclization reaction between these kinds of propiolic acid derivatives and free 2-aminopyrdines does not proceed, due to the relatively low basicity and nucleophilicity of the latter [89]. For heterocyclization to proceed, it is necessary to use an anion-form of the corresponding *N*-(pyridine-2-yl)formamide, followed by in situ deprotection [89]. Alternatively, silver triflate, as a strong aprotic Lewis acid, can be postulated to catalyze such reaction with a free form of the corresponding 2-aminopyridine [90]. Finally, the use of ethylene glycol as a solvent may also promote the reaction [91]. Therefore, such significant contradictions raise doubts on the synthetic sequences, presented in the above-mentioned patent.

After all, the starting material for the synthesis of intermediates **35** and **37** is still the same: 6-chloro-2,8-dimethylimidazo[1,2-*b*]pyridazine **20**. For the preparation of the first compound, it is introduced into the cross-coupling reaction with ethyl propiolate [31]. The preparation of the second compound is a multi-step process, finishing with the palladium-complex catalyzed carbonylation of **20**, leading to the isolation of the corresponding 2,8-dimethylimidazo[1,2-*b*]pyridazine-6-carboxylic acid [32]. The impurities, remaining from the previous synthetic transformations appear to be critical to the success of both of the methodologies.

**Figure 14:**
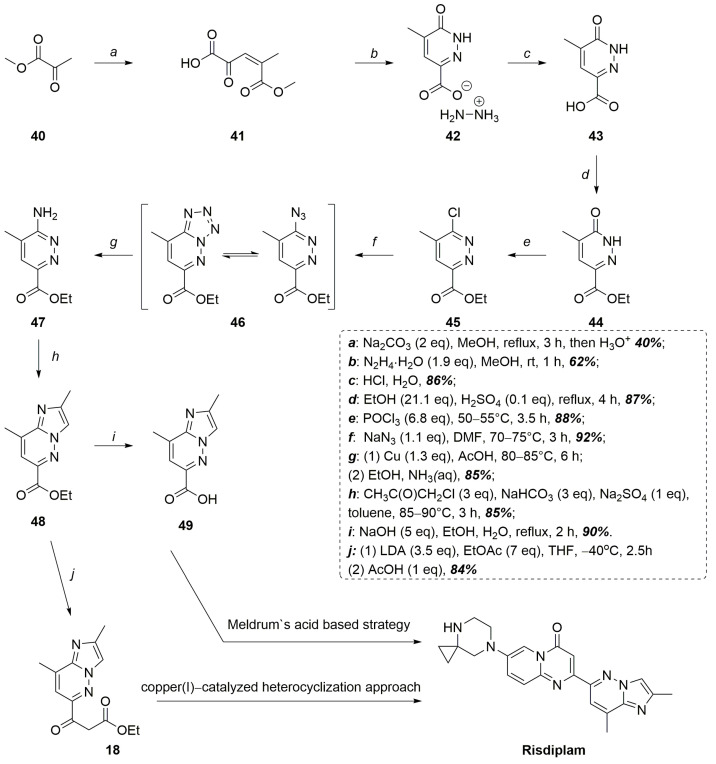
Novel, total Pd-catalyzed free approaches to the synthesis of risdiplam.

To overcome all these difficulties, our group has proposed a simple and convenient way that completely avoids the palladium-complex catalyzed reactions in the synthesis of risdiplam [38]. The key feature of it was the synthesis of 3-hydroxy-4-methylpyridazin-6-carboxylic acid **43** via the Knoevenagel condensation of methyl pyruvate, followed by heterocyclization of the crude reaction product with hydrazine. The thus obtained heterocyclic acid was subjected to esterification, deoxychlorination, and indirect amino-dechlorination yielding an ethyl ester of 3-amino-4-methylpyridazin-6-carboxylic acid **47**. Treatment of the latter with chloroacetone led to the formation of ethyl 2,8-dimethylimidazo[1,2-*b*]pyridazine-6-carboxylate **48** (Figure 14) [38].

Then, upon Claisen condensation with ethyl acetate it formed a 3-oxoester **18**, which was successively converted into the corresponding 3-aminoacrylate **16**, which is a key intermediate in the risdiplam preparation that was described earlier [39]. Alternatively, **48** may be hydrolyzed to the corresponding acid **49** and introduced into the Meldrum-acid based synthesis of risdiplam, also described previously [38]. A comprehensive head-to-head comparison of the developed routes with the originators’ optimized manufacturing process is not feasible because of substantial differences in design, logic, and numerous other important non-chemical factors. Nevertheless, a simple metric-based comparison can be performed: the optimal commercial process furnishes risdiplam in 12 steps with an overall yield of approximately 9.6%. The route featuring copper-catalyzed heterocyclization delivers the target molecule in a comparable 11 steps with an overall yield of about 10%, whereas the pathway that employs Meldrum’s acid-derived intermediates proceeds in 12 steps, (four of which are telescoped one-pot sequences), to deliver an overall yield of 17%. It is important to note that these estimates were calculated from intermediates that already incorporate the pyridazine fragment. In practice, each of the developed methodologies adds three initial steps that convert very inexpensive and simple reagents into the starting compound **43** with a modest overall yield of around 20%, thereby eliminating dependence on the commercially supplied intermediates, such as compound **23**, while enabling large-scale preparation of a previously scarce and valuable building block **43** of considerable synthetic interest (priced at US $1086 per gram).

Thus, this comprehensive review of risdiplam synthetic methodologies reveals a clear hierarchy of strategies, spanning from questionable patent claims to genuinely innovative breakthroughs. Certain reported routes—particularly those employing unprotected spirocyclic amines or unstable propiolic acid derivatives—raise serious concerns regarding feasibility and scalability. Regardless, the landscape has been dramatically reshaped by the embrace of rigorously validated methodologies. Roche’s systematically optimized commercial process stands as a benchmark in industrial process development, effectively resolving the regioselectivity and scalability challenges that hindered early discovery routes. Equally notable are the recent academic contributions that have achieved complete palladium independence while preserving synthetic efficiency, thereby demonstrating that complex heterocyclic targets can be realized via environmentally sustainable and cost-effective pathways.

## 3. Conclusions

The synthetic evolution of risdiplam represents a paradigmatic example of modern pharmaceutical process development, where academic innovation converges with industrial necessity to address complex synthetic challenges. This comprehensive analysis reveals a remarkable transformation from the initial palladium-dependent discovery routes, characterized by regioselectivity issues and scalability limitations, to sophisticated contemporary methodologies that have fundamentally redefined the synthetic landscape for complex heterocyclic targets. The successful transition from precious metal catalysis to metal-free methodologies, addresses critical industry concerns including cost containment, supply chain resilience, and environmental sustainability. The modular synthetic frameworks developed for risdiplam itself and its key intermediates provide versatile platforms for structure-activity relationship exploration and next-generation active pharmaceutical substances development. This newfound capacity to selectively sculpt each heterocyclic building block, without compromising synthetic agility, unlocks the systematic fine-tuning of pharmacological profiles for tomorrow’s drug contenders, poised to tackle not only the scourge of spinal muscular atrophy but a plethora of different ailments. The relentless march of synthetic innovation promises a future brimming with therapeutic breakthroughs. These advancements will not only address the urgent needs of today but will also fuel the creation of tomorrow’s life-saving medications, making them more readily available through streamlined production and dramatically reduced costs.

## Figures and Tables

**Table 1:** Comparison of reaction conditions and yields using different protecting groups on the 4,7-diazaspiro[2.5]octane fragment, compared with the unprotected analog.

Substrate	Substitute	Reaction Conditions	Yield (%)	Reference
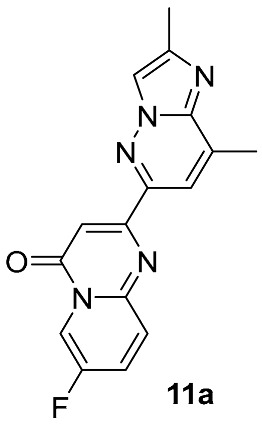	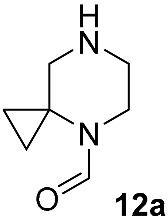	K_2_CO_3_ DMSO, 120–160 °C overnight	50	[50]
	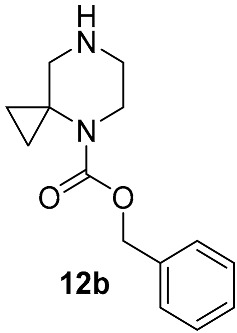	DIPEA DMSO,130 °C 48 h	47	[33]
	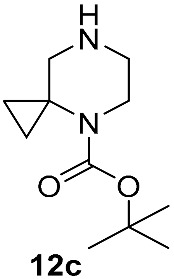	DBU DMAc,120 °C 6 h	42	[39]
	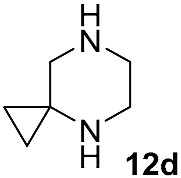	DIPEA DMSO 130 °C,48 h	18	[27]

## Data Availability

The original contributions presented in this study are included in the article. Further inquiries can be directed to the corresponding author.
